# The Portuguese adapted version of the fear of covid-19 scale for the postpartum period

**DOI:** 10.1192/j.eurpsy.2022.611

**Published:** 2022-09-01

**Authors:** D. Pereira, A.T. Pereira, B. Wildenberg, A. Gaspar, C. Cabacos, N. Madeira, A. Macedo

**Affiliations:** 1 Faculty of Medicine of University of Coimbra, Institute Of Psychological Medicine, Coimbra, Portugal; 2 Centro Hospitalar e Universitário de Coimbra, Department Of Psychiatry, Coimbra, Portugal; 3 Bissaya Barreto Maternity Hospital, Centro Hospitalar e Universitário de Coimbra, Department Of Gynecology And Obstetrics, Coimbra, Portugal

**Keywords:** Perinatal, Fear of COVID-19, Postpartum

## Abstract

**Introduction:**

The Portuguese version of the *Fear of COVID-19 Scale* (FCV-19S; Cabaços et al. 2021), composed of seven items, presented good validity and reliability to be used in general population. To be used within perinatal context, specifically in the postpartum period, we have added an item related to the baby (item 8 – “I’m afraid my baby will be infected with coronavirus-19”).

**Objectives:**

To analyze the psychometric properties of Portuguese adapted version of the *Fear of COVID-19 Scale* for the postpartum period **(**FCV-19SP), namely construct validity, internal consistency, and convergent validity.

**Methods:**

207 women (mean age= 33.51 ± 5.23 years) recruited in the postpartum period (9,06 ± 8,52 months after delivery) fill in a set of self-reported validated questionnaires: Perinatal Depression Screening Scale (PDSS), Perinatal Anxiety Screening Scale (PASS) and Coronavirus-19 Fear Scale for the postpartum period (FCV-19SP).

**Results:**

CFA revealed that the unifactorial model composed of eight items presented good fit indexes (X^2^/df=1.508; CFI=.991; GFI=.974; TLI=.983; p[RMSEA≤.01] = .049), better than those of the seven items version (X^2^/df=3.963; CFI=.957; GFI=.909; TLI=.905; p[RMSEA≤.01] =.219). Cronbach alpha for the FCV-19SPP was α=.880. The total score significantly (p<.01) and moderately correlated with PDSS (r=.262) and PASS (r=.371).

**Conclusions:**

The FCV-19SP is a valid and reliable questionnaire to assess fear of COVID-19 in women in the postpartum period.

**Disclosure:**

No significant relationships.

